# A prospective study on insect bite hypersensitivity in horses exported from Iceland into Switzerland

**DOI:** 10.1186/s13028-018-0425-1

**Published:** 2018-11-03

**Authors:** Sigurbjörg Torsteinsdottir, Stephan Scheidegger, Silvia Baselgia, Sigridur Jonsdottir, Vilhjalmur Svansson, Sigridur Björnsdottir, Eliane Marti

**Affiliations:** 1 Institute for Experimental Pathology, Biomedical Center, University of Iceland, Keldur, Keldnavegur 3, 112 Reykjavik, Iceland; 2 Department of Clinical Research & VPH, Vetsuisse Faculty, University of Berne, Länggass-str 124, 3012 Bern, Switzerland; 3 Mobile Pferdepraxis, FA Osteopathie GST, Oberdettigenstrasse 50, 3043 Uettligen, Switzerland; 4 Agricultural University of Iceland, Hvanneyri, 311 Borgarnes, Iceland

**Keywords:** *Culicoides*, Icelandic horses, Insect bite hypersensitivity, *Simulium*, Sulfidoleukotriene release assay

## Abstract

**Background:**

Insect bite hypersensitivity (IBH) is an IgE-mediated dermatitis caused by bites of *Culicoides* spp., which occurs frequently in horses imported from Iceland to continental Europe. IBH does not occur in Iceland because *Culicoides* species that bite horses are not present. However, *Simulium vittatum* (*S. vittatum*) are found in Iceland. In Europe, blood basophils from IBH-affected horses release significantly more sulfidoleukotrienes (sLT) than those from healthy controls after in vitro stimulation with *Culicoides nubeculosus* (*C. nubeculosus*) and *S. vittatum*. Aims of the study were: (I) using the sLT release assay, to test if horses living in Iceland were sensitized to *S. vittatum* and (II) to determine in a longitudinal study in horses imported from Iceland to Switzerland whether the sLT release assay would allow to predict which horses would develop IBH.

**Results:**

Horses in Iceland, even when living in high *S. vittatum* areas, were usually not sensitized to *S. vittatum* or *C. nubeculosus.* Incidence of IBH in the 145 horses from the longitudinal study was 51% and mean time until IBH developed was 2.5 ± 1 year. Before import and after the first summer following import, there were no significant differences in sLT release between the endpoint healthy (H) and IBH groups. After the 2nd summer, when the number of clinically affected horses increased in the endpoint IBH group, a significantly higher sLT release after stimulation with *C. nubeculosus* but not with *S. vittatum* was observed. After the 3rd and 4th summer, the endpoint IBH group had a significantly higher sLT release with *C. nubeculosus* and *S. vittatum* than the endpoint H group. Some of the horses that remained healthy became transiently positive in the sLT release assay upon stimulation of their peripheral blood leucocytes with *C. nubeculosus*.

**Conclusions:**

Horses in Iceland are not sensitized to *S. vittatum*. In horses that develop IBH, sensitization to *S. vittatum* is secondary to sensitization to *C. nubeculosus* and probably a result of an immunological cross-reactivity. A sLT release assay cannot be used to predict which horses will develop IBH. A transient positive reaction in the sLT release assay observed in horses that remained healthy suggests that immunoregulatory mechanisms may control an initial sensitization of the healthy horses.

## Background

Insect bite hypersensitivity (IBH) in horses also known as summer eczema (SE), sweet-itch, Queensland itch, Kasen or *Culicoides* hypersensitivity, is a chronic, recurrent seasonal dermatitis of horses caused by an allergic reaction to the bite of midges, *Culicoides* spp. (reviewed in [1]). IBH affected horses were also reported to react against other blood feeding insects like black flies (*Simulium* spp.), stable flies (*Stomoxys calcitrans*), mosquitoes and horseflies [2–4].

**Fig. 1:**
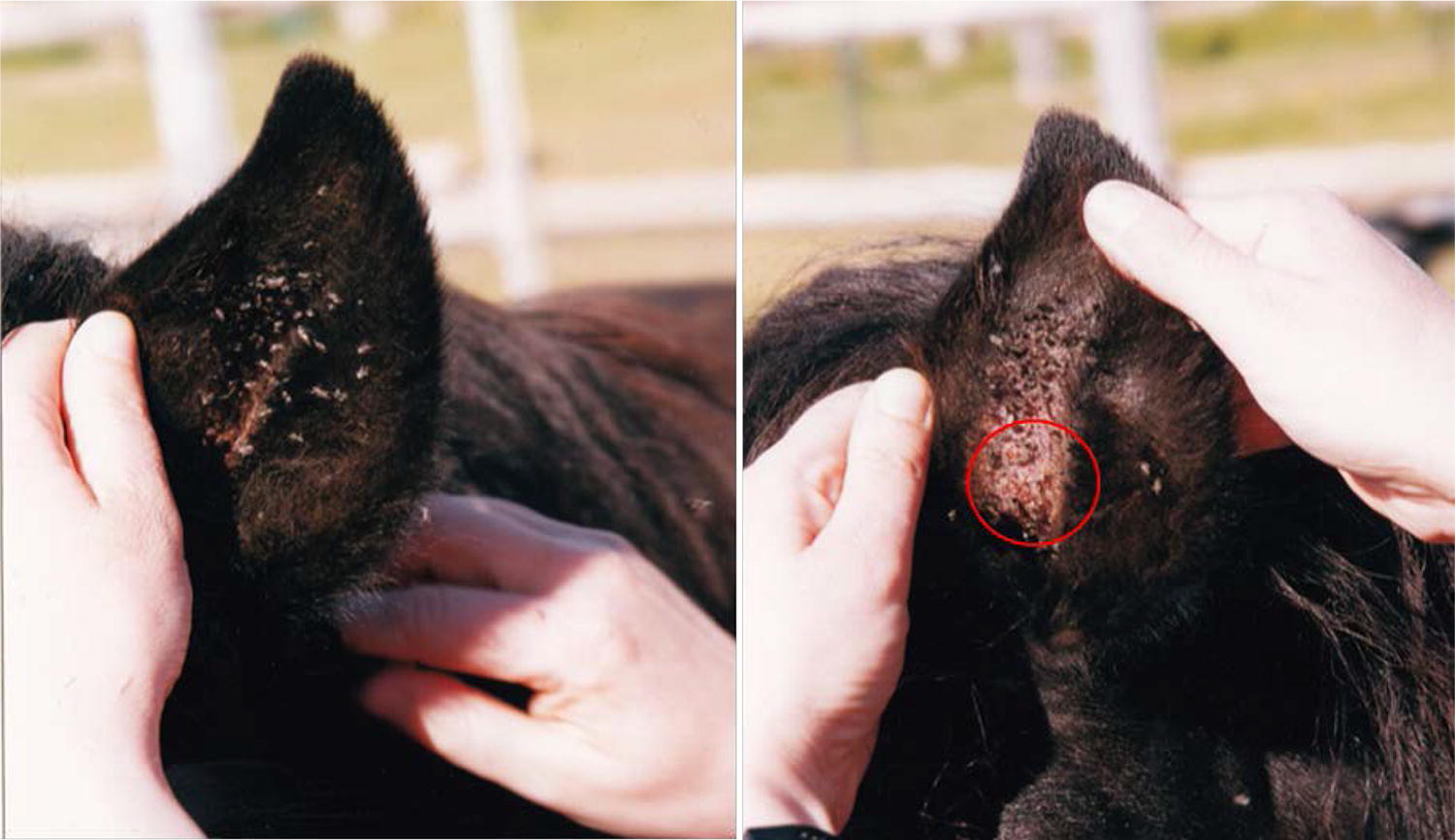
*Simulium vittatum* (black flies) in the ear of a horse in Iceland (**a**). Bleeding skin lesions (within red circle) caused by the *Simulium* bites in the ear of the same horse (**b**). The pictures were taken at the time of blood sampling in Iceland

The geographic distribution of the disease typically correlates with the location of *Culicoides* spp. but because these are not present in Iceland, Icelandic horses do not develop *Culicoides*-associated IBH. However, the black fly *Simulium vittatum* (*S. vittatum*) is indigenous to Iceland [5] and can occur in high numbers during the summer period in some areas and has been shown to feed on horses (Fig. 1). Icelandic horses that have been exported to continental Europe are predisposed to IBH, as 50% or more develop IBH within 2 years of export, if living in a known habitat of the responsible insect [6], while in Icelandic horses born in Europe the prevalence of IBH is similar to other breeds (3–10%) [7–9]. While a genetic predisposition for IBH is well-documented for horses born in an environment where *Culicoides* spp. are present [10–12] and gene regions associated with IBH have been identified in various breeds [13, 14], a genetic basis for IBH in Icelandic horses imported from Iceland to Europe could not be established [6, 10]. The fact that these horses are not exposed to *Culicoides* spp. antigens early in life, due to their absence in Iceland, is the most likely factor for the high prevalence of IBH in these horses after import [10].

Several studies have shown that IBH is an IgE-mediated, type I hypersensitivity reaction [1]. Horses with IBH but only rarely healthy control horses, have serum IgE antibodies against salivary gland proteins of *Culicoides* spp. [15–20]. Furthermore, in vitro stimulation of peripheral blood leucocytes (PBL) with *Culicoides* spp. allergens leads to the release of histamine or sulphidoleukotrienes (sLT) in IBH-affected but only rarely in healthy control horses [21–23]. The value of a sLT release assay, with *Culicoides nubeculosus* (*C. nubeculosus*) as allergen, for in vitro diagnosis of IBH has been evaluated in a well characterised, large population of IBH-affected and healthy animals and was shown to have a sensitivity of 80% and a specificity of 97% [4]. This study had also shown that horses with IBH are often not only sensitized to *C. nubeculosus* but also to *S. vittatum* [4]. These horses also have IgE antibodies binding to salivary gland allergens of both *C. nubeculosus* and *S. vittatum* [24] and at least one of the *S. vittatum* allergens is cross-reactive with the corresponding allergen from *C. nubeculosus* [25]. These findings raised the question whether horses living in Iceland could be sensitized to *S. vittatum* allergens, as these black flies are present in Iceland and bite horses, and whether horses sensitized to black flies in Iceland would be more prone to develop IBH after importation to *Culicoides*-rich environments, like Switzerland.

The aims of our study were (I) to test whether horses living in Iceland were sensitized to *S. vittatum* allergens by using the in vitro sLT release test and (II) to determine in a longitudinal study of horses imported from Iceland to Switzerland, whether the in vitro sLT release assay with *C. nubeculosus* and *S. vittatum* allergen extracts would allow any prediction whether horses would develop IBH.

## Methods

### Design of the study

Data was collected in the years 2000–2010 from 275 horses of the Icelandic breed all born in Iceland, of which 130 remained in Iceland and 145 were exported to Switzerland. The exported horses were monitored at the end of every summer after import for occurrence of clinical signs of IBH over a period of one to up to six summers. The horses classified as remaining healthy (H) had to have been exposed to *Culicoides* spp. for at least three summers. Seventy-two of the 145 imported horses arrived in winter (out of IBH season, defined as 1st of October until 31st of April) when the adult insects causing IBH are not present in Switzerland, while the remaining 73 horses were imported during the IBH season. A clinical examination was performed once a year at the end of each summer, blood samples were taken and the clinical history was recorded. Furthermore, in 40 of these horses, clinical examination and blood sampling had already been performed in Iceland before export. At the end of the study the clinical status towards IBH (healthy/IBH/unknown) was determined.

### Horses

The group of horses remaining in Iceland consisted of 91 males and 39 females with an average age of 9.5 years (range 3–25 years). Out of these 130 horses, 76 were from infested black fly areas and 54 from areas with low exposure. The group of exported horses (n = 145) consisted of 111 males and 34 females. After import the horses lived in many different stables with other Icelandic horses in Switzerland. The age of the horses at the time of export was 4–12 years.

All horses were classified at the end of each IBH season as IBH-affected, not affected with IBH (healthy) or unknown diagnosis when the clinical signs and history did not allow a determination of whether the horse had IBH or not. This was for example the case when horses had mild pruritus and were immediately treated locally with repellents or anti-inflammatory ointments and thus had no clinical signs of IBH at the time of examination.

IBH-affected horses had pruritus with scratching and scaling, excoriations and thickening of the skin along the dorsal midline, mainly at the base of the mane and tail. Sometimes the ventral midline and/or the head were affected too. When clinical signs occurred for the first time, the diagnosis was confirmed retrospectively after remission of clinical signs in winter and recurrence of the disease the next summer. Following clinical diagnosis of IBH all horses received a wide variety of treatments such as sweet itch blankets, local application of various lotions for insect and pruritus control and in few cases systemic application of the corticosteroid triamcinoloni acedonidum (Kenacort^®^). Some of the IBH-affected horses were kept in the stable most of the day or were not allowed access to pasture during the IBH season.

The H horses had no clinical signs of scratching or skin alteration and were not treated against IBH. After import, all horses were vaccinated against tetanus and equine influenza and dewormed regularly.

### Blood samples

Blood samples from the horses remaining in Iceland were obtained in August, when *S. vittatum* are active in Iceland. The horses in Switzerland were sampled at the end of each summer and 40 of them also before export. Sampling was done by jugular venipuncture into ACD-B vacuette tubes (Greiner Bio-One vacuette GmbH, St-Gallen, Switzerland). Blood samples were kept at 10–25 °C and the sLT release assay (see below) performed within 24 h.

### Clinical endpoint

The horses remaining in Iceland had no clinical signs of IBH.

Horses exported to Switzerland and assigned to the H endpoint group had not shown clinical signs of IBH at any time of the follow up study and had to be free of IBH at least three summers after import from Iceland, even though living in an environment where IBH-affected horses were living (i.e. as marker for the presence of *Culicoides* spp.). These horses had not been subjected to prophylactic treatments against IBH (for example blankets etc.).

In 21 horses the clinical outcome was not clear enough to assign them to the healthy or IBH-endpoint group. This was the case when they were treated against IBH (blanket, repellents, etc.) as soon as they were slightly itchy or had crusts at the base of the mane and tail and the owners did not want to stop this treatment to see whether clinical signs of IBH would develop.

Horses assigned to the IBH endpoint group had shown the typical clinical signs of IBH, which recurred the next summer after remission in winter.

### In vitro sulfidoleukotriene (sLT) release assay

The Equine CAST^®^ (Cellular Antigen Stimulation Test; Bühlmann Laboratories AG Schönenbuch, Switzerland) was performed as previously described [4]. Briefly: allergen extracts were made from frozen insects (whole body) [21]. *C. nubeculosus* were laboratory bred, while *S. vittatum* had been collected in Iceland. The *C. nubeculosus* extract was used at a final concentration of 2 μg/mL and the *S. vittatum* extract was used at a final concentration of 10 μg/mL [4].

The leukocyte rich plasma from the blood samples was collected, transferred into a tube and centrifuged at 130×*g* for 10 min at RT. The supernatant containing 90% of the platelets was removed and the pelleted cells were resuspended in 1 mL stimulation buffer (Bühlmann Laboratories AG) per 9 mL of ACD-B anticoagulated blood. Cell stimulation was performed following the manufacturer’s instruction, either without stimulation to determine the spontaneous sLT release, or stimulation with Concanavalin A (20 μg/mL) as a control or with the *C. nubeculosus* and *S. vittatum* extracts. After 40 min incubation at 37 °C the supernatants were removed and kept frozen for maximum 1 month until further processing. sLT determination was carried out following the manufacturer’s instructions using the Equine CAST^®^ ELISA (Bühlmann Laboratories AG). For all further evaluations the values of the net stimulation were used, i.e. the spontaneous sLT release was subtracted from the values obtained with Concanavalin A or with the allergen extracts. Horses with sLT release < 250 pg/mL with Concanavalin A were classified as ‘non responders’ (NR) [4, 21]. In the 1st year, 6 out of the 126 horses tested in the CAST^®^ were classified as NR, in the 2nd year 2 out of 111, in the 3rd year 6 out of 85 and in the 4th year, 9 out of 60. The CAST results with the insect extracts of NR horses were not included in the statistical analyses.

To distinguish between positive or negative test results the cut-off values determined previously [4] were used. For the *C. nubeculosus* stimulation, the cut-off was 310 pg/mL and for *S. vittatum* 200 pg/mL.

### Statistical analyses

Statistical analyses were carried out with the statistical software package NCSS 2011 (NCSS, Kaysville, Utah 84037, USA). A preliminary analysis indicated that the releases of sLT (pg/mL) were not distributed normally, even after logarithmic transformation. Medians and ranges were used subsequently for descriptive purposes.

To compare sLT release in the horses in Iceland living in a high or a low *Simulium* infested area, a Mann–Whitney U-test was performed.

With the 112 horses in Switzerland with a clear clinical endpoint (H or IBH) at the end of the study, we analyzed whether gender and season of import (2-tailed Fisher’s exact test), age at import, and number of summers and months until the horses developed IBH (two sample T-test) were associated with clinical disease.

The nonparametric Mann–Whitney U-test was used to compare continuous variables such as released sLT after stimulation with Concanavalin A, *S. vittatum* or *C. nubeculosus* whole body extract in IBH-affected and healthy horses.

To compare binary variables such as number of IBH-affected and healthy horses with sLT levels above or below the cut-offs defined above, the 2-tailed Fisher’s exact test was used.

The clinical diagnosis and CAST results with the insect extracts were available at all time points over a period of at least four summers in 29 endpoint H and 27 endpoint IBH horses. In order to investigate whether the CAST with the allergen extracts would allow identification of IBH prone horses before they develop clinical signs of the disease, the data were analyzed as follows: The summer when IBH-affected horses developed clinical signs of IBH for the first time was defined as T0 and the previous summers when the respective horses were exposed to insect bites but did not show clinical signs of IBH were set as T − 1, T − 2, T − 3 etc. The years following the first summer with IBH were defined as T + 1, T + 2 etc. In the horses that remained healthy until the end of the study, i.e. over four summers, T0 was defined as the last year of the study and T − 1, T − 2, T − 3 as the respective preceding summers. The percentage of horses giving a positive test results with *C. nubeculosus* or *S. vittatum* antigen extracts at the different time points was then compared between the groups of horses that remained healthy or developed IBH. The two tailed Fisher’s exact test was used to determine whether there was a significant difference in the proportion of horses giving a positive test result between both groups.

## Results

### sLT release from *C. nubeculosus* and *S. vittatum*-stimulated peripheral blood leukocytes of horses remaining in Iceland

From the 130 horses remaining in Iceland, two were NR and had thus to be removed from the analysis. Median sLT released after stimulation with *C. nubeculosus* and *S. vittatum* extracts were low and there were no significant differences between the horses living in a low (n = 53) compared to those living in a high *Simulium* area (n = 75) (Table 1). Overall, only 3% of the horses gave a CAST results above the cut off after stimulation with *S. vittatum* extract and the values were low. Only one of the horses had a positive CAST results when stimulated with *C. nubeculosus* extract.

**Table 1:** Sulfidoleukotriene (sLT) production by peripheral blood leucocytes (PBL) from horses remaining in Iceland in areas with low or high infestation with *Simulium vittatum*

Incubation of PBL with	Median (range) of sLT released (pg/mL) from PBL of horses coming from areas with		Low versus high P
	Low *Simulium* (N = 53)	High *Simulium* (N = 75)	(MWU-test^d^)
Cul2	68 (0–925)^a^	74 (0–164)	Ns
Sim10	6 (0–408)^b^	7 (0–391)^c^	Ns

PBL were stimulated with *C. nubeculosus* extract (Cul2: 2 μg/mL) or *S. vittatum* extract (Sim10: 10 μg/mL)

The cut off values defined in Baselgia et al. [4] were used

*Ns* not significant

^a^One horse above cut off (310 pg/mL)

^b, c^Two horses in each group above cut off (200 pg/mL)

^d^Mann–Whitney U test

### Development of clinical signs of IBH in horses imported from Iceland to Switzerland

**Fig. 2:**
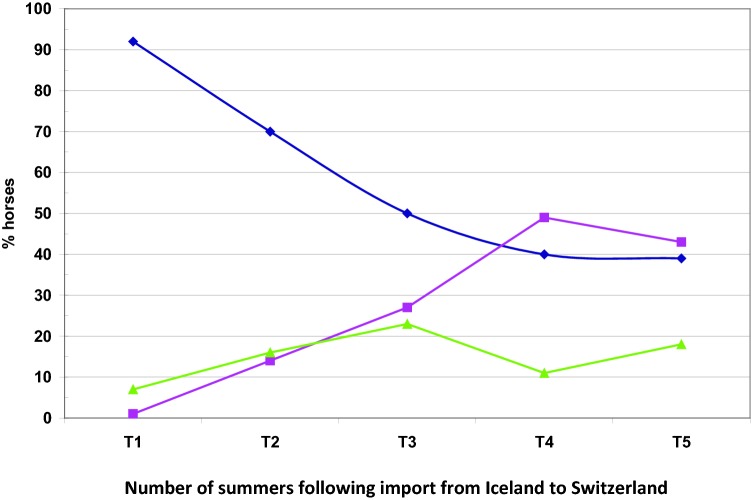
Percentage of horses that over time developed IBH (pink line, squares), remained healthy (blue line, diamonds) or were classified with unclear clinical diagnosis (green line, triangles). The total number of horses included in the study at the different times is presented in Table 2. T1 − T4 corresponds to the number of summers spent in Switzerland after importation from Iceland to Switzerland, i.e. one (T1), two (T2), three (T3) and four (T4) summers after importation to Switzerland

Before export, the horses had, as expected, no clinical signs of the disease. After the 1st summer in Switzerland 92% of the 145 horses were classified as healthy, two horses were IBH-affected and 7% could not be classified as healthy or IBH-affected. One year later, 70% of the 135 horses that could be followed were still healthy, 14% had IBH and 16% could not be classified. After the third season in Switzerland, the percentage of horses that were free of IBH decreased to 50%, while 27% of the imported horses had IBH and 23% could not be classified. After the 4th season, the percentage of IBH-affected horses was higher (49%) than the percentage of healthy horses (40%). In the remaining 11% of the horses the clinical diagnosis was unclear. In the last investigated period the percentages stayed virtually unchanged, with 43% IBH-affected, 39% healthy and 11% not classified horses (Fig. 2, Table 2).

**Table 2:** Number of horses involved in the longitudinal study before export from Iceland and one (T − 1), two (T − 2), three (T − 3) and four (T − 4) summers after importation from Iceland into Switzerland

	Iceland	T1	T2	T3	T4	T5
Clinical status at time point	40	145	135	122	78	30
Tested in CAST						
Horses that remained healthy	15	71	55	41	19	
Horses that developed IBH^a^	19	49	54	38	32	
Total	34	120	109	79	51	

^a^At some time during the study

### Factors influencing the clinical endpoint

From the 112 imported horses exposed for at least three summers to *Culicoides* spp., 55 horses (49%) had remained healthy and 57 (51%) had developed IBH. With these horses, we analyzed whether gender, age at import and season of import could influence the clinical endpoint. Furthermore, we also determined the mean numbers of summers and months following import until the horses developed IBH.

As shown in Table 3, the gender distribution was the same in the H and IBH endpoint groups, as in both the majority were males (76% and 77%, respectively). There were also no significant differences for the age at import as the horses from both groups had been imported into Switzerland at a mean age of 7.1 and 7.5 years, respectively. Fifty-six percent of the 55 endpoint H horses had been imported during the IBH season and 44% out of the IBH season. Contrarily, the endpoint IBH horses were imported more frequently out of the IBH season (54%) than during the IBH season (46%), however, this difference was not statistically significant. The horses developed IBH after a mean number of 2.8 ± 1 summers or 30 months after import and the endpoint H horses were followed in our study for a mean of 3.6 ± 0.8 summers or 39 months.

**Table 3:** Factors influencing the clinical endpoint in 55 horses that remained healthy (H) and 57 horses that developed insect bite hypersensitivity (IBH)

	Clinical endpoint		Significance	
	H (n = 55)	IBH (n = 57)	Fisher’s	P (t-test)
Gender (males:females)	42:13	44:13	Ns	
Age at import (mean year ± SD)	7.1 ± 1.55	7.5 ± 1.61		Ns
Import during the IBH season (N/%)	31 (56%)	26 (46%)	Ns	
Import out of the IBH season (N/ %)	24 (44%)	31 (54%)	Ns	
Mean number ± SD of summers since import until clinical outcome^a^	3.6 ± 0.8	2.8 ± 1		≤ 0.001
Mean number ± SD of months since import until clinical outcome^a^	39 ± 10	30 ± 12		≤ 0.001

For this part of the study, only horses that had spent three or more summers in Switzerland were included in the healthy endpoint group

^a^Defined as time until diagnosis of IBH was first made or, in the healthy group, time point until which horses were monitored without showing clinical signs of IBH

### Time course of CAST results in relation to clinical endpoint

**Fig. 3:**
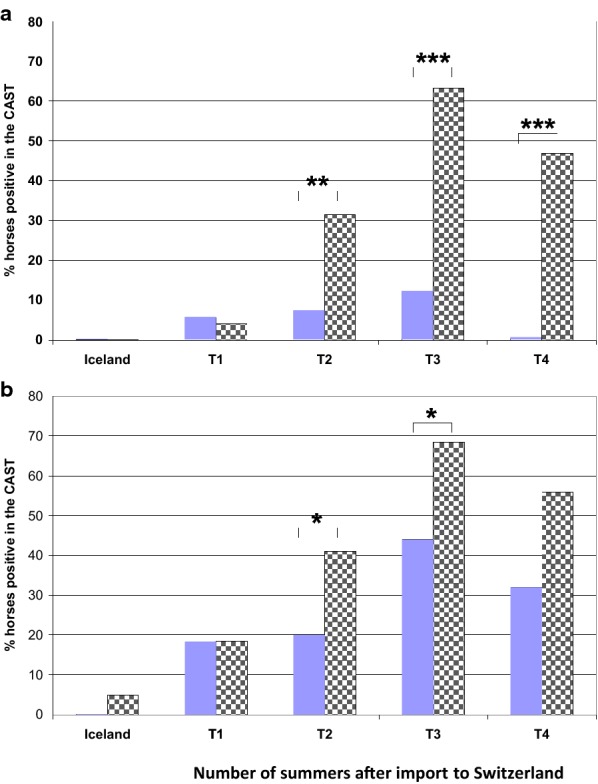
Longitudinal study of horses imported from Iceland to Switzerland. Percentage of clinical endpoint healthy (H, filled blue columns) or insect bite hypersensitivity (IBH, columns with grey squares) horses with a positive test result in the CAST with **a** *Culicoides nubeculosus* extract (2 μg/mL) or **b** *Simulium vittatum* extract (10 μg/mL), in Iceland before export to Switzerland (T_Iceland_) and one to four (T1–T4) summers after importation from Iceland into Switzerland. The number of horses tested in the CAST at the different times is given in Table 2. *P < 0.05; **0.05 > P < 0.01; ***P < 0.001

The median values of sLT (pg/mL) released after stimulation with *C. nubeculosus* and S*. vittatum* extracts at the different time points in the endpoint IBH and endpoint H groups over the duration of the study are shown in Table 4. Of the 40 horses tested in Iceland and then exported, one was positive with the *S. vittatum* extract, but the sLT release was only slightly above the cut off (275 pg/mL). This horse developed IBH in Switzerland. sLT release was low in all other horses tested in Iceland and thus there were no significant differences in median sLT release with *S. vittatum* or *C. nubeculosus* between the endpoint H and endpoint IBH groups. The same was true the first summer after import. Nevertheless, at that time single horses (6%) gave positive CAST results with *C. nubeculosus* and even more frequently with *S. vittatum* (20%). However, these differences were not statistically significant (Fig. 3). After the second summer, with the increasing number of horses developing clinical signs of IBH (Fig. 2), the median sLT release with the insect extracts started to increase in the group with IBH as clinical endpoint and became significantly different from the median of the H endpoint group for *C. nubeculosus* but not for *S. vittatum* extract (Table 4). These differences became even more pronounced after the third summer following import. Accordingly, after the second summer 31% of the horses with IBH as a clinical endpoint gave a positive CAST result with *C. nubeculosus* versus only 7% of the H endpoint group (*P *< 0.01, Fig. 3a). This difference increased after the third summer, with over 60% of the endpoint IBH horses giving a positive CAST result while the proportion of the endpoint H horses giving a positive test result with *C. nubeculosus* stayed at around 10% (Fig. 3a). A similar, although weaker difference was seen for the CAST with *S. vittatum* extract after the second (positive CAST in endpoint IBH group 41% versus 20% in endpoint H group; *P* < 0.05), and third summer (70% versus 44%, P < 0.05) following import (Fig. 3b). After the 4th summer the median sLT levels decreased in the H as well as in the IBH endpoint groups with both insect extracts (Table 4). Interestingly, within the H endpoint group there were hardly any horses that gave a positive test result with the *C. nubeculosus* extract, while about 60% of the endpoint IBH group were positive at that time point. The percentage of horses giving a positive test result also decreased with the *S. vittatum* extract, but there were still 30% of the horses in the H endpoint group that were positive with *S. vittatum* after the 4th summer.

**Table 4:** Median (range) sLT release (pg/mL) after incubation of peripheral blood leukocytes (PBL) with *C. nubeculosus* extract (Cul2: 2 μg/mL) or *S. vittatum* extract (Sim10: 10 μg/mL) from horses that remained healthy (H) or developed insect bite hypersensitivity (IBH) at different time points (see Table 2 for number of horses included at each time point)

Time point	Incubation of PBL with	Median (range) of sLT released (pg/mL) from horses with clinical endpoint		H versus IBH P
		H	IBH	(MWU-test^a^)
Iceland	Cul2	51 (0–109)	56 (0–109)	Ns
	Sim10	17 (0–91)	5 (0–275)	Ns
T − 1	Cul2	51 (0–1291)	65 (0–2763)	Ns
	Sim10	26 (0–2251)	30 (0–2411)	Ns
T − 2	Cul2	70 (0–3467)	100 (0–5900)	< 0.01
	Sim10	28 (0–4791)	85 (0–5350)	< 0.1
T − 3	Cul2	60 (0–4855)	968 (0–4827)	< 0.001
	Sim10	100 (0–4745)	718 (0–11834)	< 0.01
T − 4	Cul2	53 (0–4804)	244 (3–4798)	< 0.0001
	Sim10	53 (0–2402)	323 (0–4798)	< 0.01

^a^Mann–Whitney U test

### Predictive value of the sLT release assay for the identification of IBH susceptible horses before first clinical signs of IBH

In order to assess if the CAST could allow to predict whether a horse would develop IBH before outbreak of the clinical signs, horses were grouped in those that developed IBH and those that remained healthy and the year when the first signs of IBH occurred was set as T0 while the preceding years were set as T − 1, T − 2 etc.

**Fig. 4:**
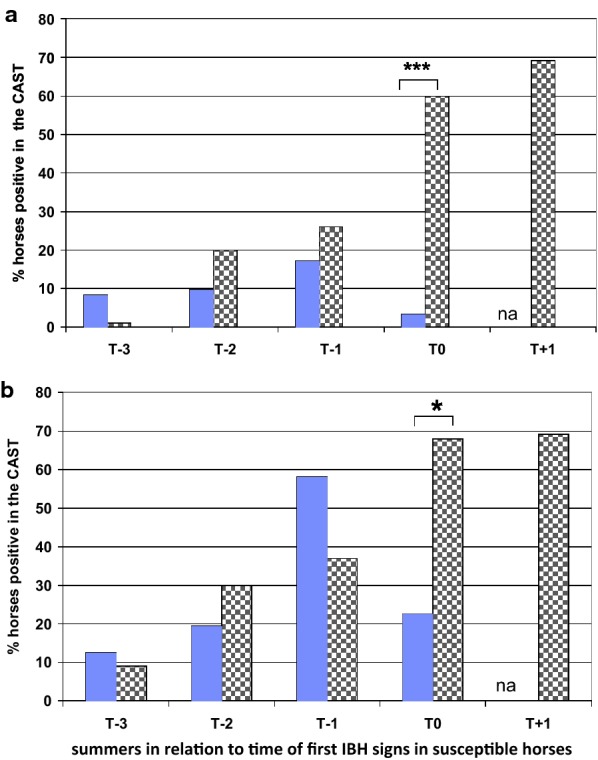
Predictive value of the CAST for the identification of IBH susceptible horses before first clinical signs of IBH. Percentage of clinical endpoint healthy (N = 29; blue filled column) or IBH (N = 27; column with grey squares) horses that gave a positive CAST result with **a** *Culicoides nubeculosus* (2 μg/mL); or **b** *Simulium vittatum* (10 μg/mL) extracts. T0 corresponds to the first summer when IBH was diagnosed, or in the healthy horses the last year of the follow up study. T − 1, T − 2 and T − 3 represent the summers preceding T0, and T + 1, the summer following the clinical diagnosis of IBH. *P < 0.01; ***P < 0.0001

Figure 4 shows that the CAST only allows the identification of horses developing IBH in the season when the disease occurs, as it is the only time when the percentage of horses with IBH and with a positive test result either with *C. nubeculosus* or *S. vittatum* is significantly different from the percentage of healthy horses positive in the CAST. At that time, 60% of the IBH horses but only 3.4% of the H horses are positive with *C. nubeculosus*. A varying proportion of horses was also positive in the CAST in the preceding years, however, a relatively high percentage of the healthy group was also positive and the differences between both groups were not significant.

## Discussion

Horses imported from Iceland to continental Europe represent a unique model to study the natural course of IBH because of the absence of *Culicoides* in Iceland and the high prevalence of this disease following import to an environment where *Culicoides* are present. One of the major difficulties of the study was that the horses were privately owned and living in different stables all over Switzerland. We tried to control for exposure to *Culicoides* spp. by only including horses living where exposure to *Culicoides* spp. was known to occur, by using the presence of other IBH-affected horses. The severity of IBH was not evaluated because horses were often treated at first clinical signs. Severe IBH cases were thus rare. The horses in Iceland showed no clinical signs of IBH and after the first summer in Switzerland, only two confirmed cases of IBH out of the 145 horses were found. The prevalence then increased to up to 50% in the fourth summer following import, similarly to what has been found previously [6]. Although only 30% of the horses had a confirmed diagnosis of IBH after the third summer, we speculate that the real incidence was already higher at that time point because in 20% of the horses the diagnosis was not clear and thus only 50% of the horses were definitely free of IBH. The mean time until onset of IBH following import was 2.5 years and comparable to what has been described [6, 26]. In accordance with a previous study [8], the incidence of IBH was higher in horses imported out of the IBH season compared to import during the IBH season but this difference was not statistically significant. Similarly to earlier publications [6, 8], we found no influence of gender or age at importation on the incidence of IBH. However, our study group only consisted of Icelandic horses exported as adults (4–12 years). The incidence of IBH in Icelandic horses exported at young age (7–10 months) is much lower than if exported as adults and is similar to that of Icelandic horses born in Europe [27]. This might be explained by a higher capability of foals to make a regulatory immune response compared to adults [28]. The age at first exposure to *Culicoides* spp. appears thus to be an important predisposing factor [1, 27], while, unlike for horses born in *Culicoides*-rich environments, genetic factors probably play a minor role [6, 10].

The CAST has been evaluated thoroughly for its use with *C. nubeculosus* and *S. vittatum* extracts to confirm a clinical diagnosis of IBH [4]. A high specificity (> 95%) of this test with *C. nubeculosus* extract with a sensitivity of 78% was found, while specificity and sensitivity were lower with *S. vittatum* extract. Our aim was to investigate whether Icelandic horses that would develop IBH following exportation to *Culicoides* spp. areas would already be positive in CAST before the first onset of clinical signs and also, whether horses sensitized to *S. vittatum* in Iceland would be more prone to develop IBH after importation to *Culicoides*-rich environments. To establish the baseline values, 128 horses that remained in Iceland and 40 horses from the follow up study were tested in the CAST in Iceland. Only one out of a total of 168 horses showed a positive test result with *C. nubeculosus*, confirming earlier studies [29, 30]. The results after stimulation with *S. vittatum* are more interesting because *S. vittatum* are the only haematophagous flies that bite horses in Iceland [5]. The *S. vittatum* extract was made from *S. vittatum* captured from the wild in Iceland. However, only four of the 128 horses bled in the *Simulium* season reacted to the extract and there were no differences between horses coming from high and low *Simulium* environment. From the 40 horses tested before export, only one was positive upon stimulation with *S. vittatum*. This horse, tested and exported at the end of summer, was positive in the CAST with both extracts a few months after arriving in Switzerland and developed clinical IBH the following summer. This suggests that the horse was already sensitized to *S. vittatum* allergens before export, but without clinical signs of IBH. In general, *Culicoides* spp. allergens seem to be needed as sensitizers whereas after sensitization many of the allergic horses cross-react to homologous proteins from *Simulium* spp. Immunological cross-reactivity is a well-known phenomenon in allergy [31]. Homologous allergens found in *Simulium* spp. and *Culicoides* spp. that share common epitopes have been documented [19, 25, 32].

The percentage of horses showing a positive CAST with *C. nubeculosus* increased over time and got significantly higher in the endpoint IBH compared to the H group after the second summer following import, reaching a peak after the third summer with 63% of the endpoint IBH and 12% of the endpoint H horses being positive. After the 4th summer the percentage of positive horses decreased to 50% in the endpoint IBH group, probably because most IBH-affected horses were treated, i.e. exposure to the allergens was strongly reduced. The results of the CAST could not be evaluated in all horses at all time points because blood samples were not always available and because of non-responders [4, 21]. A complete data set over four summers was thus only available for 29 and 27 endpoint H and IBH horses, respectively. With these horses we investigated whether horses that developed IBH gave a positive CAST result in the years preceding first clinical signs. Twenty-six percent and 20% of these horses were positive in the CAST with *C. nubeculosus* 1 and 2 years before clinical IBH, respectively. Nevertheless, these results were not useful to identify IBH predisposed horses because 8–17% of the endpoint H horses were also positive in the test. However, most of the horses of the H endpoint group became negative in the following years, while 60% of the IBH group was positive in the CAST in the year when clinical signs first occurred and this percentage increased to nearly 70% 1 year later (Fig. 4). At all time points there were more horses positive with *S. vittatum* than with *C. nubeculosus* and at T − 1 there were more endpoint H than IBH horses positive in CAST with this allergen. With both insect extracts a significant difference between the H and IBH endpoint group was thus only obtained when the horses become clinically affected with IBH. The proportion of healthy horses positive in the CAST with *C. nubeculosus* was higher than what was found previously in a study where only horses living for many years in Switzerland were included (17% versus < 5%; [4]). We do not know why a relatively high proportion of the H endpoint horses were positive in the CAST. Interestingly, most of them became CAST negative again later on. This suggests that a majority of horses become sensitized to these insects to some degree but that regulatory mechanisms may then control sensitization [33, 34].

## Conclusions

The major findings of this study are that 50% of Icelandic horses imported from Iceland develop IBH following import into Switzerland and that exposure to insect bites such as *Culicoides* spp. for at least one but usually for two to three summers is required to develop IBH. This indicates that horses in Iceland are not sensitized against *Culicoides* spp. or *Simulium* spp., although the latter are native to Iceland, a result that was confirmed by the CAST. Importantly, the present study shows that a positive CAST result when stimulating with *C. nubeculosus* in horses recently imported from Iceland can only be used to confirm clinical cases of IBH. The CAST should not be used to identify horses predisposed to IBH before occurrence of first clinical signs, because a part of the horses that remain free of this disease can be transiently positive in the test. This suggests that horses can be sensitized to *Culicoides* spp. allergens to some degree but that regulatory mechanisms may then control further development of the disease.
